# Severe Renal Colic as a Trigger of Coronary Thrombosis on Plaque Erosion

**DOI:** 10.1016/j.jaccas.2026.108607

**Published:** 2026-05-30

**Authors:** Xue Gong, Minghui Xue, Lifang Zhao, Jing Yang

**Affiliations:** Department of Cardiology, Shanghai Xuhui Central Hospital, Zhongshan-Xuhui Hospital, Fudan University, Shanghai, China

**Keywords:** acute myocardial infarction, optical coherence tomography, plaque erosion, renal colic, stress trigger

## Abstract

**Background:**

Acute myocardial infarction may result from the complex interplay between transient systemic triggers and vulnerable coronary substrates.

**Case Summary:**

A 37-year-old man presented with persistent chest pain after severe renal colic. Electrocardiography demonstrated lateral ST-segment elevation with diffuse ST-segment depression. Emergency coronary angiography revealed thrombotic occlusion of the proximal left anterior descending artery. Optical coherence tomography (OCT) identified extensive thrombus overlying a mildly atherosclerotic segment without evidence of plaque rupture, consistent with probable plaque erosion. Thrombus aspiration followed by drug-coated balloon angioplasty restored TIMI flow grade 3 without stent implantation.

**Discussion:**

This case illustrates a possible trigger-substrate interaction, in which severe visceral pain may act as a systemic trigger for coronary thrombosis in a patient with an erosion-prone phenotype. OCT was pivotal in defining the underlying mechanism and guiding a stent-free strategy.

**Take-Home Messages:**

Transient systemic stressors may act as triggers of acute coronary events. OCT enables mechanism-based, stent-free management in selected cases of plaque erosion.

## Visual Summary

**Figure:**
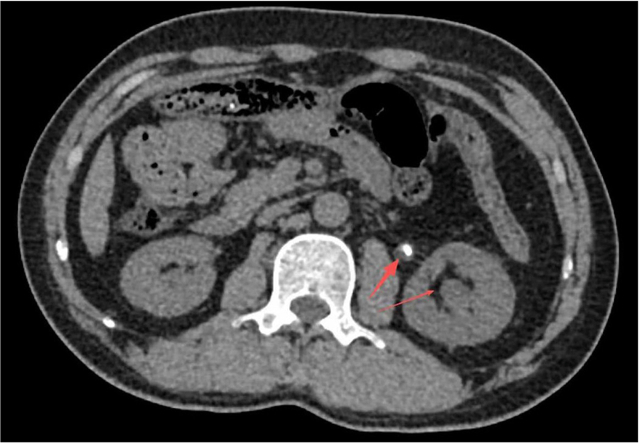

## History of Presentation

A 37-year-old male courier presented to the urology emergency department with 6 hours of severe left-sided renal colic, followed by 2 hours of persistent retrosternal chest pain accompanied by nausea, vomiting, and diaphoresis. On examination, the patient exhibited acute distress due to pain. His blood pressure was 152/94 mm Hg, heart rate 108 beats/min, and oxygen saturation 98% on room air. Left costovertebral angle tenderness was noted.

Emergency computed tomography confirmed a 0.6-cm calculus in the upper left ureter with associated proximal hydroureter and hydronephrosis (Figure 1). Despite treatment with antispasmodics, the chest pain persisted. Initial electrocardiogram demonstrated sinus tachycardia with mild ST-segment elevation (0.5-1.5 mm) in leads I and aVL, accompanied by diffuse ST-segment depression (2-3 mm) across the inferior and precordial leads (Figure 2).

**Figure 1:**
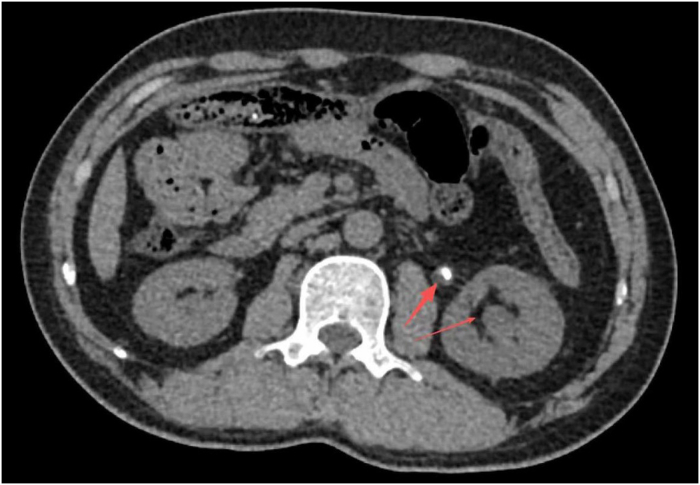
Computed Tomography of the Urinary Tract Computed tomography imaging demonstrates a 0.6-cm calculus located in the upper segment of the left ureter (white highlight), associated with proximal hydroureter (thick red arrow) and hydronephrosis (thin red arrow), confirming the diagnosis of renal colic.

**Figure 2:**
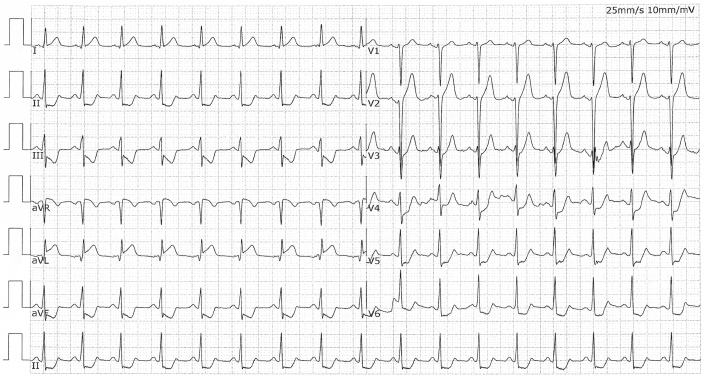
Electrocardiogram on Admission The electrocardiogram demonstrates sinus tachycardia with mild ST-segment elevation in leads I and aVL, accompanied by diffuse ST-segment depression in the inferior leads and across the precordial leads.

## Past Medical History

The patient had a 3-year history of ureteral calculus with 2 prior acute episodes, but no history of chest pain. He also had a significant history of heavy smoking and chronic sleep deprivation. There was no known history of cardiovascular disease or relevant family history.

## Differential Diagnosis

The initial differential diagnosis included acute coronary syndrome (ACS), vasospastic angina, myocarditis, and noncardiac chest pain.

## Investigations

The patient was transferred to our chest pain center, where repeat electrocardiogram confirmed persistent lateral ST-segment elevation and diffuse ST-segment depression. Laboratory testing revealed elevated cardiac biomarkers (cardiac troponin I: 0.168 ng/mL, creatine kinase–MB: 9.94 ng/mL, myoglobin: 586.88 ng/mL). Given the high suspicion of ST-segment elevation myocardial infarction (STEMI), the catheterization laboratory was activated immediately.

## Management

Emergency coronary angiography demonstrated complete occlusion of the proximal left anterior descending artery (LAD), with a heavy thrombus burden (Figure 3A). After manual thrombus aspiration, partial flow restoration revealed a residual ∼70% stenosis in the mid-LAD (Figure 3B). Optical coherence tomography (OCT) identified a large intracoronary thrombus within the proximal LAD, partially obscuring visualization of the underlying plaque (Figure 4A). No clear evidence of plaque rupture, significant lipid burden, or calcification was identified in the adjacent segments (Figure 4B).

**Figure 3:**
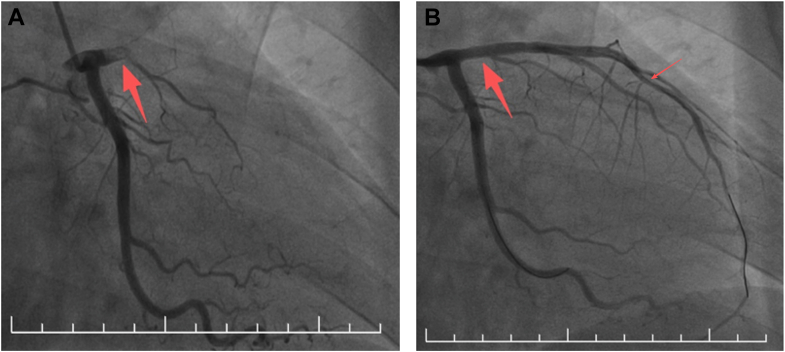
Coronary Angiography Findings (A) Emergency coronary angiography demonstrates complete occlusion of the LAD with a large thrombus burden (thick red arrow). (B) Final angiography after thrombus aspiration and drug-coated balloon angioplasty shows restoration of coronary flow with minimal residual stenosis (thick red arrow) and approximately 70% residual stenosis in the mid-LAD (thin red arrow). LAD = left anterior descending artery.

**Figure 4:**
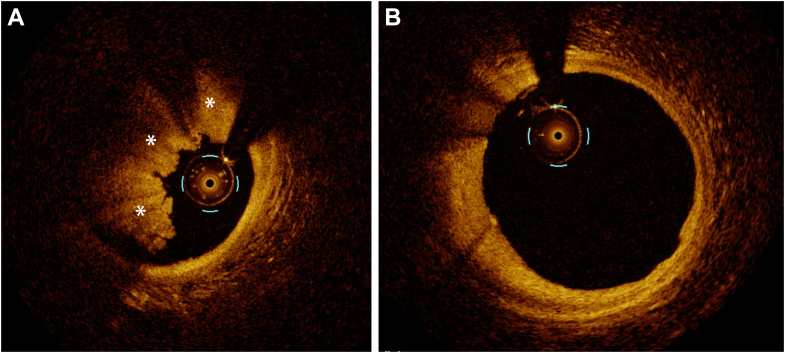
Optical Coherence Tomography Findings (A) OCT reveals erythrocyte-rich thrombus (white asterisks) in the proximal LAD, partially obscuring visualization of the underlying plaque. (B) No superficial lipid or calcification is observed immediately proximal or distal to the thrombus site. LAD = left anterior descending artery; OCT = optical coherence tomography.

A stent-free strategy was adopted. Thrombus aspiration followed by drug-coated balloon (DCB) angioplasty restored TIMI flow grade 3, with no obvious residual stenosis in the proximal lesion. The residual ∼70% mid-LAD stenosis was managed conservatively with planned follow-up angiographic and intracoronary imaging reassessment. The patient received intensive lipid-lowering and guideline-directed medical therapy.

## Outcome and Follow-Up

After the procedure, the patient remained free of recurrent chest pain, although intermittent renal colic persisted. Echocardiography revealed hypokinesis of the interventricular septum, apex, and anterior wall, with a reduced left ventricular ejection fraction of 40%. Laboratory testing showed elevated N-terminal pro–B-type natriuretic peptide (2,194 pg/mL) and inflammatory biomarkers (C-reactive protein: 13.55 mg/L, interleukin-6: 17.90 pg/mL, tumor necrosis factor-α: 19.76 pg/mL).

Treatment included dual antiplatelet therapy (DAPT), statins, and guideline-directed medical therapy. Subsequently, the patient successfully underwent elective endoscopic lithotripsy for his ureteral stone after an abbreviated course of DAPT.

## Discussion

We describe a young patient presenting with STEMI shortly after an episode of severe renal colic. OCT demonstrated a large thrombus overlying a mildly diseased coronary segment without evidence of fibrous cap rupture, consistent with probable plaque erosion.^1^ These findings highlight the importance of intracoronary imaging in defining the underlying mechanism of ACS.

ACS is increasingly recognized as the result of interactions between underlying plaque vulnerability and transient systemic triggers.^2^^,^^3^ Severe renal colic represents an intense nociceptive stressor that may lead to renal and systemic sympathetic activation and catecholamine surges.^4^ The resulting hemodynamic perturbations, increased coronary shear stress, and prothrombotic milieu may collectively facilitate thrombus formation on an erosion-prone plaque.^4^^,^^5^

The patient exhibited several features consistent with a plaque erosion–prone phenotype reported in prior studies,^6^^,^^7^ including young age, heavy smoking, and elevated inflammatory markers despite limited underlying atherosclerosis. OCT findings of extensive thrombus without fibrous cap disruption further supported this mechanism, highlighting the trigger-plaque interaction in the pathogenesis of the acute event.

A key strength of this case is the use of intracoronary imaging to guide a mechanism-based revascularization strategy. OCT enabled accurate differentiation of plaque erosion from rupture, thereby supporting a stent-sparing approach.^8^ Although current evidence does not support the routine use of DCB in ACS, this strategy may offer potential advantages in selected patients, particularly in younger patients with erosion-prone lesions and limited atherosclerotic burden.^9^ Avoidance of permanent metallic implants may reduce long-term risks, such as in-stent restenosis and late device-related events, while preserving future revascularization options. In the acute setting, dynamic factors including vasoconstriction and a heavy thrombus burden may limit angiographic assessment and stent optimization,^9^ and patient preference may also favor a stent-sparing approach. In this context, OCT plays a critical role in guiding lesion-specific, individualized interventional decision-making.

In the present case, OCT-guided management enabled a stent-free strategy, with DCB angioplasty performed at the proximal culprit segment after thrombus modification. The residual mid-LAD stenosis was deferred for staged reassessment. Notably, the stent-free strategy also allowed for a shorter DAPT duration, facilitating the patient's subsequent endoscopic lithotripsy for his ureteral stone.

## Conclusions

This case demonstrates that ACS may be precipitated by transient systemic stressors, such as severe visceral pain, in the presence of a susceptible coronary substrate. It underscores the importance of maintaining clinical vigilance when chest pain occurs in atypical contexts. More broadly, this case illustrates the value of integrating pathophysiological understanding with intracoronary imaging to enable mechanism-informed, individualized management of ACS.

**Visual Summary:**
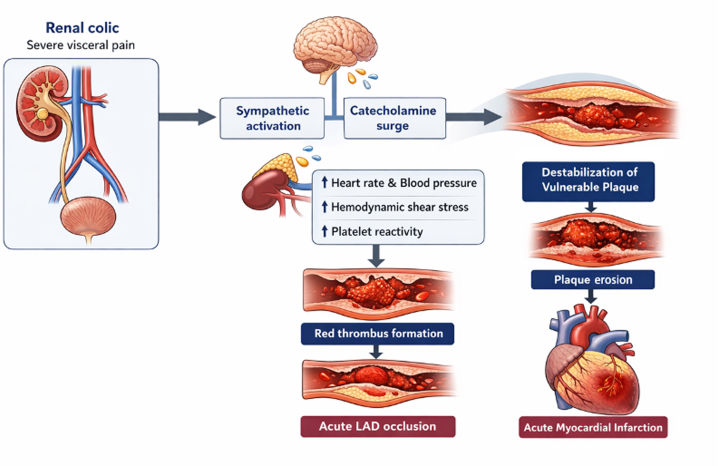
Proposed Trigger–Coronary Substrate Interaction in Plaque Erosion–Related Thrombosis This schematic illustrates the proposed interaction between a transient systemic trigger and an underlying vulnerable coronary substrate in this case. Severe renal colic may induce sympathetic activation, catecholamine release, and hemodynamic stress, contributing to a transient prothrombotic state. In parallel, the patient exhibited features consistent with a plaque erosion–prone phenotype, including young age, smoking, and mild plaque burden. The interaction between these factors may have facilitated thrombus formation on an intact fibrous cap without plaque rupture, as demonstrated by OCT. This imaging finding supported a stent-free strategy, including thrombus aspiration and drug-coated balloon angioplasty at the proximal lesion, with deferred evaluation of a nonculprit mid-LAD stenosis. LAD = left anterior descending artery; OCT = optical coherence tomography.

## Funding Support and Author Disclosures

The authors have reported that they have no relationships relevant to the contents of this paper to disclose.

**Take-Home Messages**

• Transient systemic stressors may act as triggers of acute coronary events.
• Optical coherence tomography enables mechanism-based, stent-free management in selected cases of plaque erosion.

## References

[1] Kolte D., Yonetsu T., Ye J.C. (2021). Optical coherence tomography of plaque erosion: JACC focus seminar part 2/3. J Am Coll Cardiol.

[2] Wereski R., Kimenai D.M., Bularga A. (2022). Risk factors for type 1 and type 2 myocardial infarction. Eur Heart J.

[3] Chan B., Buckley T., Tofler G.H. (2023). Emotional stress and physical exertion as triggers of acute myocardial infarction. Am J Cardiol.

[4] Sandrini L., Ieraci A., Amadio P. (2020). Impact of acute and chronic stress on thrombosis in healthy individuals and cardiovascular disease patients. Int J Mol Sci.

[5] Araki M., Yonetsu T., Kurihara O. (2021). Circadian variations in pathogenesis of ST-segment elevation myocardial infarction: an optical coherence tomography study. J Thromb Thrombolysis.

[6] Fang C., Dai J., Zhang S. (2019). Culprit lesion morphology in young patients with ST-segment elevated myocardial infarction: a clinical, angiographic and optical coherence tomography study. Atherosclerosis.

[7] Gulati R., Behfar A., Narula J. (2020). Acute myocardial infarction in young individuals. Mayo Clin Proc.

[8] Araki M., Park S.J., Dauerman H.L. (2022). Optical coherence tomography in coronary atherosclerosis assessment and intervention. Nat Rev Cardiol.

[9] Durand R., O'Callaghan D., Coughlan J.J. (Published online March 25, 2026). Drug-coated balloon angioplasty for coronary and peripheral artery disease: latest evidence and clinical indications. Nat Rev Cardiol.

